# PreImplantation factor (PIF) detection in maternal circulation in early pregnancy correlates with live birth (bovine model)

**DOI:** 10.1186/1477-7827-11-105

**Published:** 2013-11-15

**Authors:** Sivakumar Ramu, Christopher Stamatkin, Leo Timms, Marshall Ruble, Roumen G Roussev, Eytan R Barnea

**Affiliations:** 1 CARI Laboratories, 233 E. Erie Street, #520, Chicago, Illinois 60611, USA; 2 Department of Animal Science, Iowa State University, 2229 Lincoln Way, Ames, IA 50011, USA; 3 Bulgarian Academy of Sciences, 73 Tzarigradsko Shosse, Sofia, Bulgaria; 4 Society for the Investigation of Early Pregnancy (SIEP), 1697 Lark Lane, Cherry Hill, NJ 08003, USA; 5 BioIncept LLC, 1697 Lark Lane, Cherry Hill, NJ 08003, USA

**Keywords:** PreImplantation factor (PIF), Pregnancy BioMarker, ELISA, Live birth, Pregnancy outcome, Monoclonal anti-PIF antibody, Embryo viability

## Abstract

**Background:**

Early identification of viable pregnancy is paramount for successful reproduction. Detection of specific signals from pre-implantation viable embryos in normal pregnancy circulation would indicate initiation of embryo-maternal interaction and create a continuum to accurately reflect embryo/fetal well-being post-implantation. Viable mammalian embryos secrete PreImplantation Factor (PIF), a biomarker which plays key, multi-targeted roles to promote implantation, trophoblast invasion and modulate maternal innate and adaptive immunity toward acceptance. Anti-PIF monoclonal antibody (mAb-based chemiluminescent ELISA) accurately detects PIF in *singly cultured embryos media* and its increased levels correlate with embryo development up to the blastocyst stage. Herein reported that PIF levels (ELISA) in early *maternal serum* correlate with pregnancy outcome.

**Methods:**

Artificially inseminated (AI) blind-coded Angus cattle (N = 21-23) serum samples (day10,15 & 20 post-AI) with known calf birth were blindly tested, using both non-pregnant heifers (N = 30) and steer serum as negative controls. Assay properties and anti-PIF monoclonal antibody specificity were determined by examining linearity, spike and recovery experiments and testing the antibody against 234 different circulating proteins by microarray. Endogenous PIF was detected using <3 kDa filter separation followed by anti-PIF mAb-based affinity chromatography and confirmed by ELISA and HPLC. PIF expression was established in placenta using anti-PIF mAb-based IHC.

**Results:**

PIF detects viable pregnancy at day 10 post-AI with 91.3% sensitivity, reaching 100% by day 20 and correlating with live calf birth. All non-pregnant samples were PIF negative. PIF level in pregnant samples was a stringent 3 + SD higher as compared to heifers and steer sera. Assay is linear and spike and recovery data demonstrates lack of serum interference. Anti-PIF mAb is specific and does not interact with circulating proteins. Anti-PIF based affinity purification demonstrates that endogenous PIF is what ELISA detects. The early bovine placenta expresses PIF in the trophoblast layer.

**Conclusion:**

Data herein documents that PIF is a specific, reliable embryo-derived biomarker conveniently detectable in early maternal circulation. PIF ELISA emerges as practical tool to detect viable early pregnancy from day 20 post-AI.

## Background

In successful mammalian pregnancies, embryo-maternal interaction develops immediately post-fertilization, even before implantation occurs. This is evidenced by the decrease in circulating murine platelets observed within a few hours post-fertilization [1-3].

Our focus in this context is to identify an embryo-specific signal that conditions maternal response pre-implantation and throughout viable pregnancy. This embryo-maternal dialogue is paramount for both at initiation and correlate with successful reproductive outcome. We reported that a peptide, PreImplantation Factor (PIF) (MVRIKPGSANKPSDD) is secreted by singly cultured, human, bovine and murine embryos [4-8]. Levels in embryo culture media could be detected using a sensitive anti-PIF monoclonal antibody based mass spectrometry and ELISA [6,8]. In singly cultured bovine and murine embryos increased levels of PIF in the media correlate with viable embryos development. In contrast, PIF is absent in non-viable embryos [6]. Further, PIF is up-taken by viable bovine embryos and when added to singly cultured embryos it promotes their development [6,7]. PIF plays an essential role in human pregnancy, as it primes the endometrium for implantation, promotes trophoblast invasion and regulates systemic immune response [8-11]. In the context of recurrent pregnancy loss (RPL) PIF has two demonstrated beneficial effects: acting as a rescue factor to negate patients’ embryo-toxic serum thereby preventing embryo demise and reducing systemic NK cells cytoxicity [7,12]. We reported that PIF is present in maternal circulation, and is expressed by the human placenta and fetus [4,5,13-15]. Relevant to PIF’s immune regulatory features, translational aspects to treatment of non-pregnant autoimmune and transplantation models were documented [16-19].

Our goal herein is to establish whether early detection of PIF in maternal circulation correlates with embryo viability and favorable reproductive outcome. PIF detection in maternal circulation, rather than in embryo culture media, would substantially broaden its potential use as viability biomarker.

Bovine pregnancy provides a good model for examining PIF in this context for multiple reasons. For one, bovine and human gestation length is similar. Further, artificial insemination (AI) is conducive to documenting the time of conception and collection of timed serum sampling. Moreover, in the case of bovine pregnancy, PIF testing may have significant utility in overcoming current industry limitations in early viable pregnancy diagnosis. Rectal palpation based diagnosis is made only at >30 days post-AI. While ultrasound can be used earlier (at >25 days), 10-15% of cases visualize a non-viable embryonic sac [20,21]. Color doppler at day 20 diagnoses non pregnant cows with 74% accuracy [22]. Progesterone levels decline prior to estrus [23,24]. Placental glycoproteins, and pregnancy proteins (60, SBU-3 antigen, protein B and DG29) detect pregnancy at ~30 days, however they may also be detected post-partum [25-29]. Circulating nucleic acids can indicate pregnancy at day 20 post AI with 75% accuracy and InterferonTau induced neutrophil genes at day 21 [30-32]. Early cow pregnancy diagnosis is critical due to the high loss rate at <16 days post-AI [33,34].

The objective of the present study was therefore to apply the same PIF ELISA (previously used to identify viable cultured bovine embryos) in order to detect PIF in early bovine pregnancy serum and correlate its presence with successful calf birth. Equally significant, we aimed to show lack of PIF indicates negative pregnancy status. Serum samples were collected from Angus cattle at 10–20 days post-AI, assayed for PIF, and followed until viable birth, while non-pregnant heifers and steer served as negative controls. Furthermore, anti-PIF antibody was used to determine presence of PIF in bovine placenta as a possible source for post-implantation PIF in circulation. In aggregate, the data generated herein establishes PIF as a valuable, embryo-derived signal whose presence in early circulation indicates pregnancy status earlier and more reliable than other methods.

## Methods

### Antibodies and reagents for the PIF ELISA

Biotin-conjugated mouse anti-PIF mAb, (BioIncept LLC, proprietary) horseradish peroxidase (HRP)-conjugated streptavidin (UltraAvidin-HRP), antigen-coating buffer and tetramethylbenzidine (TMB) substrate were all obtained from Leinco Technologies, Inc. (Saint Louis, MO). SEA BLOCK blocking buffer was obtained from Thermo Scientific (Waltham, MA). LumiGLO Peroxidase Chemiluminescent substrate was obtained from KPL, Inc. (Gaithersburg, MD). Synthetic 15-Amino Acid PIF peptide (BioIncept LLC, proprietary) and ovalbumin-conjugated PIF* were produced by Bio-Synthesis Inc. (Lewisville, TX). The sulfuric acid stop action solution and fish gelatin were procured from Sigma-Aldrich Co. (St Louis, MO). The LUMITRAC 600 flat-bottomed white polystyrene plates used for the chemiluminescent assay were purchased from Greiner Bio-One (Monroe, NC).

### Bovine serum samples used to detect PIF

Serum samples were collected from 21–23 multiparous dairy Angus beef cows following AI and generously provided by M. Ruble and L. Timms (Iowa State Univ. Ames, IA). All samples were run blindly. These samples were serially collected on days 10 (N = 23), 15 (N = 21) and 20 (N = 23) post-AI and the serum was separated and frozen immediately. (Two of the day 15 samples were not available for analysis). Subsequently, ultrasound was performed on day ~28 to assess viability and pregnancy and then cows were followed until calving. Once successful viable delivery was accomplished, previously collected serum samples were analyzed for PIF. In parallel, serum samples from a group of 30 known non-pregnant heifers were collected to use as *non-pregnant* controls. Steer sera were also procured from Animal Technologies, Inc. (Tyler, TX) for use as *negative* control and also to prepare standard diluent. Before testing, samples were thawed, aliquoted and coded. Serum samples underwent no more than one freeze-thaw cycle during the storage period before thawing for analysis.

### Chemiluminescent PIF ELISA

The chemiluminescent ELISA as validated in bovine embryo culture media was previously reported [6]. (BioIncept, LLC, Proprietary) Briefly, Ovalbumin-conjugated PIF was diluted in 1X antigen-coating buffer (Leinco Technologies, MO) to a concentration of 100 ng/mL. One hundred microliters of this coating solution was added to LUMITRAC polystyrene plates and incubated overnight at room temperature. Plates were then washed with PBS-T and then blocked with 300 μL/well of SEA BLOCK blocking buffer for 2 hrs at 37°C. The plates were then washed, air dried, and either used immediately or kept at 4°C in an airtight bag for up to 14 days. All serum samples were run in triplicate. In addition a number of pregnant serum samples were diluted neat, 1:2, 1:4 and 1:8 and tested to determine the optimal dilution for PIF detection.

**Figure 1:**
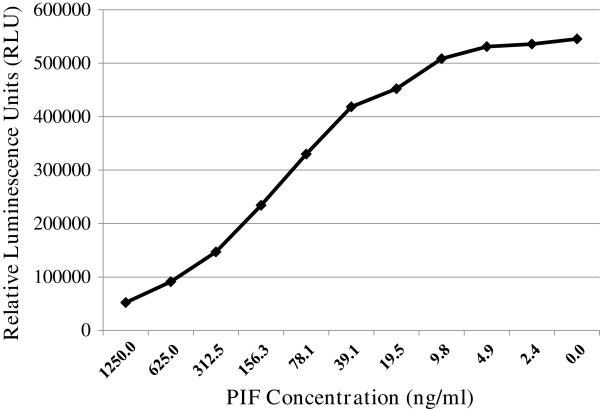
**Standard curve used for competitive ELISA for detecting PIF.** The chemiluminescent standard curve was generated by plotting the relative luminescence units (RLU) against the PIF concentration, yielding a typical sigmoidal curve.

50 μL of PIF standard was prepared in steer serum (0–1250 ng/ml) or test serum samples (diluted 1:8 in PBS-T), added to appropriate wells and the location marked using a plate layout. To this, 50 μL of biotin-conjugated anti-PIF mAb (25 ng/mL in PBS-T) was added to all wells, except the blank. Plates were then incubated for 2 hrs at room temperature. Following incubation, wells were washed four times with PBS-T and then 100 μL of UltraAvidin-HRP, diluted to 50 ng/ml in PBS-fish gelatin (1%) was added to each well and incubated for 30 min at 37°C. Plates were washed four times in an automated ELISA plate washer and 100 μL of LumiGLO Chemiluminescent substrate was then added to each well. After 5 min, the plates were read at 470 nm, with 0.5 sec integration time for each well in a SpectraMax L microplate luminometer (Molecular Devices, Sunnyvale, CA). PIF ELISA was further optimized using a checkerboard titration method. A representative standard curve (Figure 1) shows that PIF level is accurately detected at low analyte concentrations.

### Determine PIF-ELISA linearity at various serum dilutions and recovery following PIF spike

Since concentrations of PIF in a given sample may greatly vary assay linearity determination at various serum dilutions was carried out. Linearity is defined relative to the calculated amount of analyte based on the standard curve, not relative to the raw absorbance measurements. If the linearity is maintained over a wide range of dilutions, then the assay method provides great flexibility to assay samples containing different PIF concentrations. Three different concentrations, high, medium and low levels of PIF were spiked in steer sera and diluted two-fold serially. The experiment was repeated three times and the mean percent recovery was calculated using chemiluminescent ELISA, and quantified [6]. This documented that the PIF can be detected at a wide range of concentrations and the serum used does not interfere with PIF detection.

### Establish specificity of the anti-PIF mAb

The specificity and possible cross-reactivity of the anti-PIF antibody were tested using a commercially available human protein array from Ray Biotech, Inc., Norcross, GA (The RayBio Human Protein Array Product No. PAH-G1) following manufacturer protocol by RayBiotech custom service itself. Briefly, a total of 234 human recombinant or native proteins were spotted in triplicate onto the surface of a solid support (glass slide). A set of biotin-conjugated proteins produced positive signals, which were then used to identify their orientation and to compare the relative expression levels among the different wells. The biotin-labeled anti-PIF mAb, diluted 1:20,000 (the same dilution used for the chemiluminescent PIF ELISA) (described below) was then used to probe the array and bound proteins were detected using streptavidin-conjugated Cy3 Fluor dye and <1% binding was considered non-specific binding. This enabled determination of anti-PIF- monoclonal antibody’s specificity.

### PIF-based affinity chromatography use for native PIF isolation and testing by ELISA and HPLC

Serum from pregnant Angus cows, control heifers and steers were concentrated using a Centricon 3000-dalton cutoff filter (Millipore, Billerica, MA) following manufacturers protocol. This was based on consistent observation [8] that all PIF activity is observed at the <3 KDa fraction. The anti-PIF mAb was immobilized to AminoLink Plus Coupling Resin (Pierce Biotechnology Inc., Rockford, IL) to create an affinity column. The eluted <3 kDa fraction was passed through the affinity column. Subsequently, an aliquot of the purified samples were again tested by PIF ELISA to confirm the validity of the affinity purification. Purified samples were also run through HPLC (System Gold HPLC, 24 Karat software, and 250 mm × 4.6 mm, 5 μm, 120 Å, C-18 Ultrasphere Column, Beckman Coulter, Inc., Fullerton, CA). These experiments enabled HPLC confirmation of the difference in retention times in the affinity purified samples.

### Evaluate PIF expression using anti-PIF-monoclonal antibody-based immunohistochemistry (IHC) in the placenta

Early pregnancy cow placental tissues were obtained from a commercial abattoir (Animal Technologies, Inc. Tyler, TX). Tissue was immediately frozen and sent on dry ice to Zyagen Inc. San Diego Ca for IHC analysis. Briefly, samples were dissected into 1 cm × 1 cm size and fixed for 24 hrs in 10% neutral buffered formalin. Next, samples were exposed to a 30% sucrose solution overnight at 4°C and then placed in OCT solution VWR (Westchester, PA) and frozen at −20°C. Samples were then sectioned at a thickness of 7 μm and sections mounted and fixed on glass slides.

For IHC, slides were treated with cold acetone for 5 min at −20°C (to enhance the permeability), air dried for 15 min, washed for 5 min with 1X PBS, treated with 0.2% Triton X-100 in PBS for 10 min and washed with 1x PBS for 5 min. To block endogenous peroxidase activity, slides were treated with 3% hydrogen peroxide in methanol for 10 min and then washed with 1X PBS for 5 min.

The blocked sections were treated with 10% donkey serum in PBS for 1 hr before decanting the solution. The primary mouse Anti-PIF mAb was incubated for 2 hrs, at 1–200 μg/ml concentration. Biotinylated Donkey Anti-mouse secondary antibody in PBS was incubated for 1 hr followed by a wash for 5 min with 1X PBS. The slides were then incubated with Avidin-Peroxidase conjugate solution in PBS for 30 min. The samples were incubated for 10 min in DAB substrate, (Vector labs, catalog number SK-5100, Burlingame, CA) with nickel (to increase the sensitivity). Finally, the samples were washed for 5 min each with 50%, 70%, 95% and 100% ethanol, followed by xylene for 5 min (two changes) and then covered with a xylene-based mounting medium with a coverslip for microscopic examination. As negative controls, sections were processed in the absence of primary antibody, in the presence of non-immune immunoglobin or in the presence of primary antibody neutralized by an excess of peptide. This anti-PIF antibody based detection enabled documentation of PIF expression in the placenta.

### Data analysis to establish PIF levels correlation with pregnancy outcome

The chemiluminescent ELISA data was analyzed using the SoftMax Pro software (Molecular Devices) following a four-parameter logistic curve fitting, from which the concentrations of unknown were derived and adjusted for dilution.

A mean + 3SD (standard deviation) of the PIF concentrations (based on background absorbance values) in the control heifer and steer samples was used as threshold. The use of a +3SD provides a high 99.74%, stringent evaluation of PIF presence in normal pregnancy. 2 tail t test was used to analyze individual levels, where P < 0.05 was considered significant.

## Results

### PIF ELISA detects pregnancy in 100% at day20 post-AI and PIF levels correlate with live birth

#### All negative controls test PIF negative

In this study we have only used bovine serum samples which assured that all underwent synchronization defining the time of ovulation and insemination. Insemination was performed only once and otherwise there was no potential for later mating.

**Figure 2:**
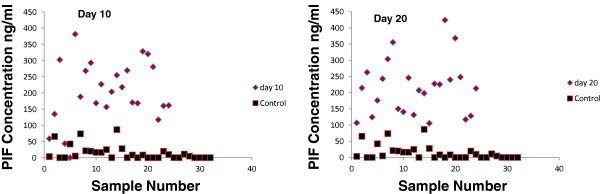
**PIF levels, day 10- post AI vs. control and day-20 post AI vs. control.** PIF levels were determined by ELISA at different days after AI in cows with documented calf delivery. Levels were compared with non-pregnant never inseminated animal. A stringent +3SD threshold (99.74%) was used vs controls. As expected, non-pregnant control PIF levels overlap with a small number of day-10 post-AI subjects, whereas by day-20, there is clear stratification.

At day-10, 22/23 (95.6%) serum cow samples were blindly diagnosed as pregnant using PIF ELISA (mean + 2SD) and subsequently calved. When using a more stringent criteria, (mean +3SD) as a threshold cut-off for non-pregnant cows only 21/23 (91.3%) were correctly identified as pregnant. One cow tested by PIF as non-pregnant at day 10; however by day 15 and 20 post-AI she was detected to be pregnant by ELISA. At day 15 18/21 (85%) serum samples were correctly diagnosed as pregnant. Remarkably, at day 20, 23/23 (100%) were detected correctly. As negative controls, 30 heifers (never inseminated or mated) and two steer samples were used. All control samples tested negative as compared with PIF positive samples (Figure 2). PIF concentrations in pregnant samples serum range was (175–227) ng/ml at day 10–20 post-AI and no significant changes in the mean concentration were found at the tested days. Background of PIF assay in non-pregnant animals is presented mean+/−SD (14+/−22) (Additional file 1: Figure S1). The data illustrates the quartiles of the individual values at background, day 10 and day 20. The median concentrations increased, and the lowest levels at day 20 were much higher than that observed at day 10. Thus PIF detection in early pregnancy circulation correlates with live calf birth.

### PIF detection is linear at different dilutions and at different spiked PIF concentrations: serum does not interfere with PIF detection

One important element in assay performance is specific detection of low analyte concentration significantly higher than the assay background. This was accomplished using the +3SD as threshold. Using different dilutions of sera with spiked PIF documented accurate detection at neat and up to 1:8 dilution of the sample (Table 1).

**Table 1:** PIF detection by ELISA

**Dilution factor (DF)**	**Observed (ng/ml)**	**Expected ng/ml**	**Recovery %**
	**X DF**	**(neat value)**	
**High spike concentration (n = 3)**			
Neat	570	625	91.2
1;2	633		101.3
1;4	615		98.3
1;8	571		91.3
**Medium spike concentration (n = 3)**			
Neat	154	156	98.7
1;2	143		91.5
1;4	139		88.9
1;8	149		95.2
**Low spike concentration (n = 3)**			
Neat	71	78	91.0
1;2	69		88.9
1;4	74		95.2
1;8	68		87.2

ELISA is linear at different dilutions and spiked PIF recovery is >90%. Observed values were assessed relative to the assay standard curve produced by a PIF standard prepared in the steer sera.

To rule out any serum interference with PIF, “spike and recovery” experiments were performed, at low, mid and high concentration (Table 1). Data showed that recovery of PIF was >90% in all concentration ranges while the blank value was zero. This indicated that serum does not interfere with PIF detection. Having previously shown the specificity of the PIF ELISA in embryo culture media, we now show that PIF ELISA in serum is equally reliable as well.

### Anti-PIF antibody is highly specific: does not interact with circulating proteins

**Figure 3:**
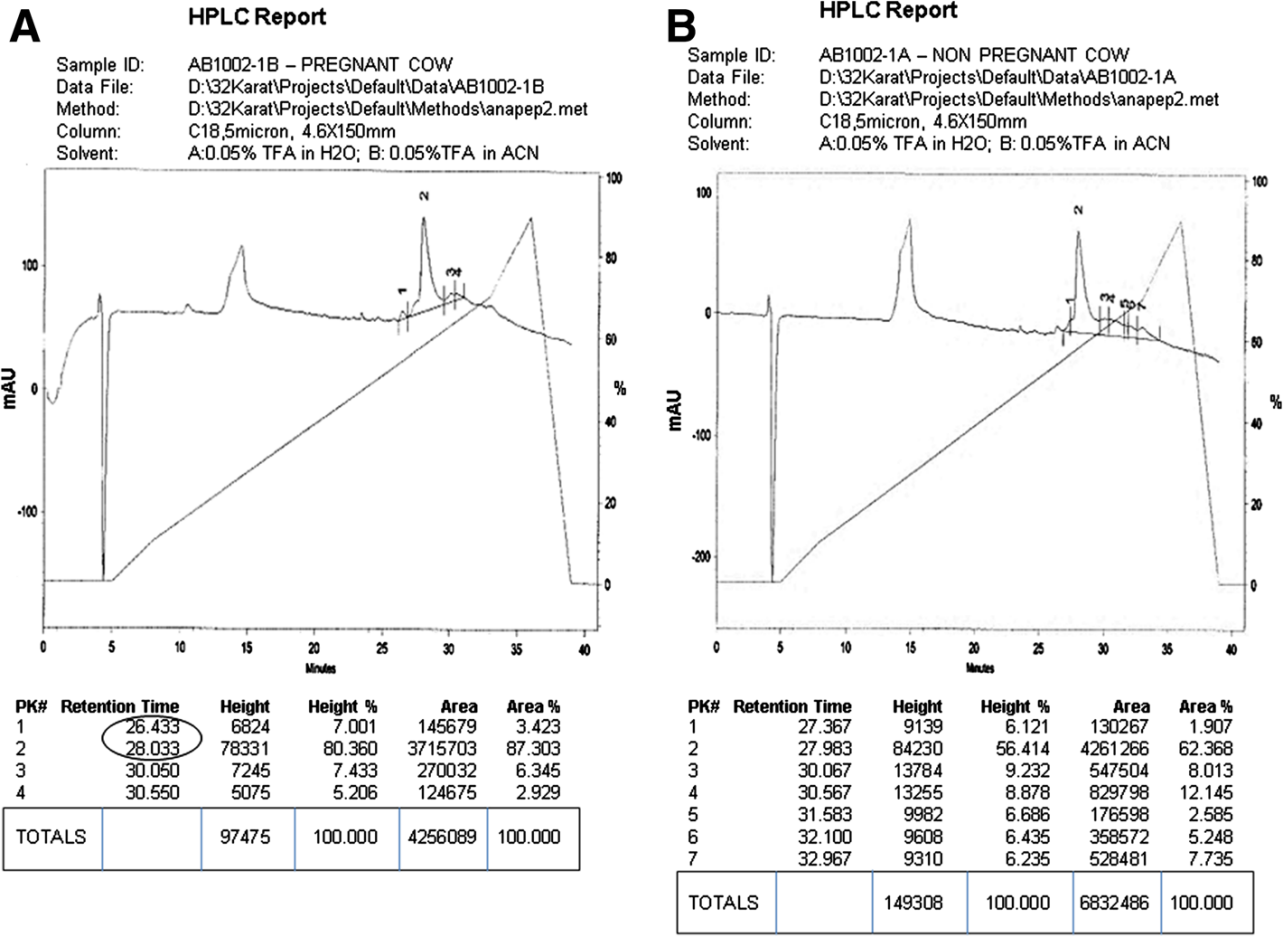
**HPLC profile of PIF in pregnant sera.** The retention time of PIF is shown in sera from **(A)** a pregnant cow, but not **(B)** in steer sera.

In contrast to embryo culture media where PIF was accurately detected by ELISA and correlated with pregnancy outcome, in maternal circulation we face a large number of proteins that could be interacting with the antibody in sera and thus interfere with the assay. To confirm the anti-PIF antibody specificity, an array of 234 circulating proteins to determine cross reactivity with the monoclonal antibody was used. The antibody did not interact with 232/234 99.1% of evaluable proteins. (Figure 3) Weak reactivity was detected with [Chemokine (C-C motif) ligand 15 (CCL15) (macrophage-inflammatory protein (MIP 1δ)] and tissue inhibitor of metalloproteinase 3 (TIMP3). Spiking protein (MIP 1δ) into sera samples did not affect ELISA sensitivity (data not shown), while the (TIMP3) interaction with anti-PIF mAb on the array was low, six fold lower than (MIP 1δ), reflecting a non-specific interaction-which actually is lower than the background. The individual antibody specificity data is presented in Additional file 2: Table S1. Thus, we demonstrate that anti-PIF mAb is highly specific.

### Accurate detection of endogenous PIF in pregnant sera by ELISA after anti-PIF mAb based affinity chromatographic separation: HPLC confirmation

To determine whether the antibody used is specific and accurate to detect endogenous PIF in serum samples, we have performed multistep PIF isolation and confirmed by ELISA. Five AI-bred pregnant cows samples were pooled and passed through an <3 kDa filter followed by an anti-PIF mAb based affinity column. The collected elute of native PIF was tested by ELISA which confirmed endogenous PIF detection. In contrast, the heifer and steer samples processed in the same manner had no PIF activity.

**Figure 4:**
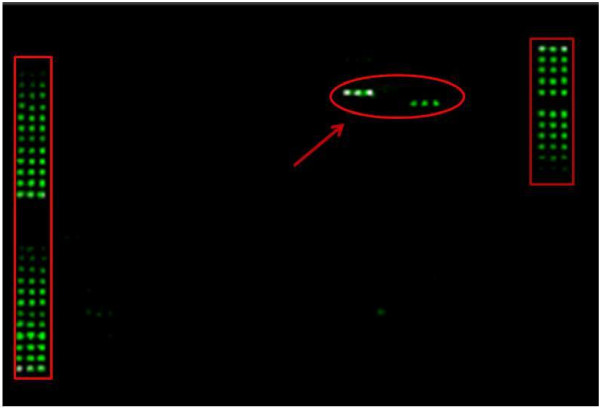
**The PIF mAb is specific.** Biotin-labeled mouse, anti-PIF mAb was screened against 243 circulating human proteins using an array. PIF mAb did not react with any of the proteins except two, (left side) MIP-1 Delta and (right side) TIMP3, circled by arrow, which were <1% as compared with controls. The biotin labeled and spotted proteins act as positive control (highlighted in two rectangles on either side of the array).

The detection of endogenous PIF was also examined by using <3 kDa filter and anti-PIF affinity chromatography followed by HPLC. The collected samples were injected into reversed phase HPLC (Figure 4). The pregnant sample profile showed a shorter retention time as compared with the steer samples. Overall such data indicates that PIF ELISA detects endogenous PIF similarly to that present in embryo culture media.

### PIF source in post-implantation gestation is found also in the placenta

Once we found that PIF is detected in maternal circulation, it was of interest to explore whether the placenta provides an additional source of PIF after implantation occurs. The placental sample analyzed was from an early gestation. Using anti-PIF monoclonal antibody based IHC, endogenous PIF was found to be expressed mostly in the trophoblastic layer. Since a sandwich ELISA method was used (as opposed to direct detection), this required a rather high antibody concentration to be used for staining. The effect was dose dependent.- the most intense staining was noted at the highest antibody concentration. The use of nickel metal enabled significant enhancement of the PIF staining (Additional file 3: Figure S2). In contrast, the same samples tested against the mouse isotype antibody (control) were negative reflecting detection specificity. Endogenous circulating PIF is likely secreted by the placenta thus allowing its measurement in maternal circulation.

## Discussion

We have previously established that PIF is an essential embryo-derived signal present in singly cultured viable post-IVF embryos *culture media* (bovine) [6]. Herein, we provide evidence in the same species using a stringent threshold, that PIF presence in maternal serum correlates with pregnancy outcome and predicts whether pregnancy will be successful. We also provide equally significant utility, confirming a lack of pregnancy in serum found to be PIF-negative (less than threshold level). Using the convenient bovine model, PIF ELISA at 10 days post-AI predicts live birth with >91% accuracy and this reaches 100% by day 20 post AI. This provides strong evidence that embryo-derived PIF signaling presence in maternal circulation is an indicator of embryonic health and a potent predictor of pregnancy outcome. We furthermore report that while prior to implantation PIF is secreted by the embryo, the placenta adds a major source of post-implantation PIF, enabling serum-based detection. PIF is hereby established as an accurate biomarker of early cattle gestation.

A number of steps were carried out to establish the PIF ELISA validity. Assay linearity and spike and recovery experiments confirmed assay precision in ligand detection. The multistep PIF isolation from pregnant serum documented detection of endogenous PIF by ELISA. The generated HPLC profile further confirmed differences between pregnant and non-pregnant samples. Anti-PIF-antibody testing against large number of circulating proteins documented target specificity. It has to be noted that specificity was directed against human proteins; therefore, interaction of anti-PIF antibody with bovine specific proteins cannot entirely be excluded. However, since in spike and recovery experiments PIF added to the serum was mostly recovered, if there is interference it is only minor and should not impair the assay performance. These overall data support the view that the ELISA used to detect PIF in bovine embryo culture media identifies a highly similar biomarker in maternal circulation [6].

Our study aimed to define an assay’s sensitivity and specificity at the earliest stages of gestation therefore blinded synchronized mating enabled exact timing of sample collection. The identical cow population and same number of cows serially tested at day 10 and 20 post-AI allowed evaluating accurately sequentially calf delivery rates by direct comparison between the two time points.

Also, since PIF ELISA was shown highly effective to diagnose controls as non-pregnant (heifers and steer) this assay could be used to document failed AI even before cows would have already returned to estrus. Further studies should examine whether PIF ELISA can also predict pregnancy loss in cows since PIF was shown instrumental to predict and prevent RPL based pathologies in women [7,12].

Presence of PIF in cow placenta confirmed our earlier data which demonstrated the same expression in human placenta and fetus [5]. The earlier data was generated by using affinity-purified polyclonal antibody while in this study the monoclonal antibody was used for staining. This further confirms expression of PIF in the placenta and helps explain the origin of circulating peptide later in pregnancy.

The PIF ELISA is a rapid and simple-to perform serum assay with high sensitivity and specificity and which currently takes only ~3 h to perform. The built-in software in the plate reader used in this study allows for immediate and precise data output. Therefore, adaptation to cow-side diagnostics is highly feasible once confirmed in larger studies.

Pertinent to PIF biomarker in cows at 10 day post-fertilization, the embryo is free and not attached to the uterus. Therefore, the PIF signal identified in maternal circulation is confirmed as purely embryonic. By day 20 the embryo has reached the uterine cavity. However only by day 33 attachment to the uterus takes place and by day 42 implantation is firmly established [35].

The study is limited since it was retrospective in nature and two samples at day 15 were not available for analysis. Also larger numbers of animals are required in order to document PIF effectiveness as a clinical tool. However, the retrospective approach was deliberate since, in absence of another marker that can detect very early pregnancy, we serially tested only those animals which successfully ended with a live birth. This was compared to data with established non-pregnant controls. Further strength of the study is assay validation analyzing antibody characteristics. Finally detection of PIF in cow placenta provides evidence that post-implantation is a source of circulating PIF.

## Conclusions

PIF ELISA accurately documents pregnancy outcome shortly post-AI, by day 20, it correlates 100% with favorable pregnancy outcome. Absence of PIF correlates at 100% with a non-pregnant status. The placenta serves as an important added source of PIF post-implantation, enabling detection in maternal serum. The observations herein have wider translational implications for PIF as a pregnancy diagnostic beyond bovine. Since bovine and human pregnancy length is highly similar (250–280 days), lessons learned from one species could be valuable for the other. We herein strengthen PIF’s premise that shortly post-conception, pregnancy outcome status can be defined and PIF presence or absence is a critical element in this equation correlating with gestation success or failure. Our ongoing multicenter human clinical trials address PIF detection and correlation of its levels with pregnancy outcome both in embryo culture media and serum (Clinical trials.gov).

## Competing interests

PIF* is a proprietary compound owned by BioIncept, LLC. E.R. Barnea, MD FACOG is its (uncompensated) Chief Scientist. CARI laboratories received an unrestricted grant from BioIncept. All authors declare no conflict of interest.

## Authors’ contributions

SR, CS and RGR performed PIF testing. LT and MR provided time samples. ERB discovered PIF, developed protocol. ERB, together with RGR and SR analyzed the data and wrote the manuscript. All authors read and approved the final manuscript.

## Supplementary Material

Additional file 1: Figure S1 PIF levels expressed as quartiles. Description: The PIF assay background was compared to levels found at day 10 and 20 post-AI. Data shows that Mean + 3SD background levels are significantly lower than PIF levels detected at day 20 post-AI. Click here for file

Additional file 2: Table S1 Testing of anti-PIF-monoclonal antibody specificity against 234 different circulating proteins. Description: Anti-PIF monoclonal antibody was tested against 234 different proteins comparing binding characteristics with His-Tag used as control. Individual data is presented showing that except for MIP-1d which was only 22% higher than the control. All other values were lower than the positive control. Click here for file

Additional file 3: Figure S2 PIF source in cow placenta. Description: The expression of PIF in first trimester cow placental tissue samples was analyzed using immunohistochemistry at different magnifications. Anti-PIF-mAb binding was tested at different concentrations, 50–200 μg/ml and results were compared to samples tested with an antibody against a mouse non immune serum (negative control). Results show that optimal anti-PIF-mAb (50 μg/ml) detects PIF within the placenta and the effect was dose dependent (a,b,c) in contrast to control antibody that failed to bind (d). Click here for file
