# Supplementary figures and images for: PreImplantation factor (PIF) detection in maternal circulation in early pregnancy correlates with live birth (bovine model)
Source: Reprod Biol Endocrinol. 2013 Nov 15;11:105. doi: 10.1186/1477-7827-11-105 (PMC3842769; doi:10.1186/1477-7827-11-105)

Three Box Plots

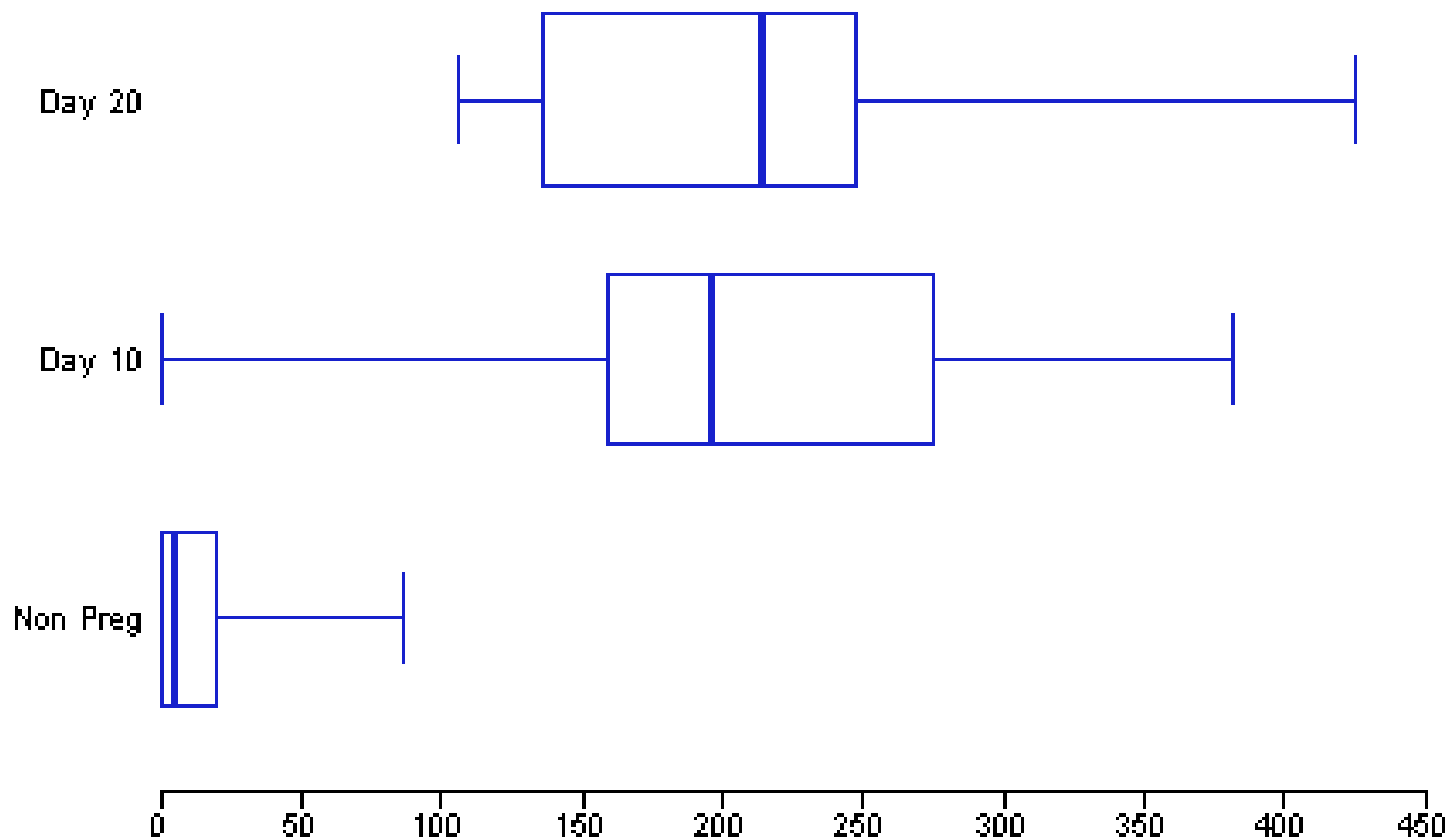

Supplement: Additional file 1: Figure S1 — PIF levels expressed as quartiles. Description: The PIF assay background was compared to levels found at day 10 and 20 post-AI. Data shows that Mean + 3SD background levels are significantly lower than PIF levels detected at day 20 post-AI. [file 1477-7827-11-105-S1.pdf]
