# Supplementary material for: PreImplantation factor (PIF) detection in maternal circulation in early pregnancy correlates with live birth (bovine model)
Source: Reprod Biol Endocrinol. 2013 Nov 15;11:105. doi: 10.1186/1477-7827-11-105 (PMC3842769; doi:10.1186/1477-7827-11-105)
Supplement: Additional file 2: Table S1 — Testing of anti-PIF-monoclonal antibody specificity against 234 different circulating proteins. Description: Anti-PIF monoclonal antibody was tested against 234 different proteins comparing binding characteristics with His-Tag used as control. Individual data is presented showing that except for MIP-1d which was only 22% higher than the control. All other values were lower than the positive control. [file 1477-7827-11-105-S2.pdf]

RayBio® Human Protein Array
Positive Control Normalization without Background

	PIF	HIS-tag
**Positive Control**	1,347,376	1,347,376
**Neg**	3	9
**4-IBBL**	75	50
**4-IBBR**	11	1
**Acrp30**	15	53,640
**Activin A**	16	47
**ALCAM**	16	53,505
**Angiogenin**	7	1
**Angiopoietin-1**	8	24,580
**Angiopoietin-2**	81	31,403
**Angiostatin**	1	7,159
**AR (Amphiregulin)**	2	1
**ART**	14	6,490
**AXL**	5	53,397
**b NGF**	189	82
**B7-1 (CD 80)**	11	18,842
**BCAM**	9	40,689
**BDNF**	2	1
**BLC**	6	6
**BMP-4**	17	423
**BMP-5**	2	7
**BMP-6**	7	15
**BMP-7**	5	4
**BTC**	5	1
**Cardiotrophin-1 (CT-1)**	10	13
**Cathepsin S**	9	7,370
**CCL21 / 6Ckine**	8	29
**CD14**	9	97
**CD27**	5	53,660
**CD30**	22	53,540
**CD40**	6	53,608
**CD40 Ligand**	15	129
**CK beta 8-1 / CCL23**	61	18
**CNTF**	4	1
**Complement Factor D**	622	53,610
**C-peptide**	28	1
**CTACK**	88	846
**CTLA-4**	46	51,528
**CXCL-16**	75	43,529
**DAN**	24	89
**DKK-1**	31	4,788
**DKK-4**	75	4,095
**DR 6**	34	265
**Dtk**	9	53,500
**E-Cadherin**	3	53,600
**EGF**	12	199
**EGF-R**	7	7
**ENA-78**	5	1
**Endoglin**	3	23
**Endostatin**	6	22
**Eotaxin**	2	1
**Eotaxin-2**	2	2
**Eotaxin-3**	13	94

**ErbB3**	16	53,681
**Erythropoietin R**	1	8
**E-selectin**	22	44
**Fas**	6	2,376
**Fas Ligand**	4	59
**Fc Gamma RII B/C (CD32b/c)**	15	53,495
**FGF-4**	1	15
**FGF-6**	9	1
**FGF-7**	6	1
**FGF-9**	33	48
**FGF-b**	9	5
**FIT-3 Ligand**	7	1
**Follistatin**	4	15
**Fractalkine**	2	6
**GCP-2**	6	29
**GCSF**	4	3
**GDNF**	5	102
**GH**	15	10
**GITR**	6	184
**GITR Ligand**	9	20,933
**GLP-1 (Glucagon Like)**	3	5
**GM-CSF**	7	16
**gp130**	9	53,655
**GRO-a**	15	18
**GRO-b**	3	5
**GRO-r**	6	9
**HB-EGF**	15	36
**HCC-4**	10	25
**HGF**	90	132
**HVEM**	21	53,607
**I-309**	6	1
**ICAM-1**	5	1
**ICAM-2**	7	12,592
**ICAM-3**	11	53,589
**IFN-gamma**	6	79
**IGF-1R**	19	43
**IGFBP-1**	2	1
**IGFBP-2**	2	1
**IGFBP-3**	3	1
**IGFBP-4**	1	1
**IGFBP-6**	6	3
**IGF-I**	9	23
**IGF-II**	2	27
**IL-1a**	10	1,484
**IL-1b**	9	27
**IL-1ra**	3	33
**IL-1RI**	7	18
**IL-1RII**	2	16
**IL-2**	4	19
**IL-2R alpha**	2	63
**IL-2R beta**	12	884
**IL-2R gamma**	1	9
**IL-3**	5	18
**IL-4**	1	3
**IL-5**	4	2
**IL-5 R alpha**	1	341
**IL-6**	2	1

**IL-6 sR**	30	28
**IL-7**	20	22
**IL-8**	7	1
**IL-9**	12	1
**IL-9 R**	10	22,881
**IL-10**	7	35
**IL-10 R alpha**	19	6,234
**IL-10 R beta**	9	53,578
**IL-11**	6	111
**IL-12p40**	1	2,250
**IL-12p70**	1	23
**IL-13**	16	42
**IL-13 Ra1**	5	53,671
**IL-13 Ra2**	22	53,651
**IL-15**	3	22
**IL-16**	7	4
**IL-17**	6	69
**IL-17B**	5	1
**IL-17C**	9	1
**IL-18 BPa**	8	153
**IL-18 R alpha**	7	34
**IL-18 R beta**	15	151
**IL-21R**	11	46
**IL-28A / IFN-lambda**	9	53,469
**Insulin**	2	6
**Insulin-Receptor**	9	4,012
**IP-10**	68	55
**I-TAC**	19	85
**LAP**	10	4
**Leptin (OB)**	52	1,172
**Leptin R**	7	11,110
**LIF**	1	14
**Light**	24	2,008
**L-selectin**	4	1
**Lymphotactin**	4	1
**MCP-1**	1	163
**MCP-2**	7	1
**MCP-3**	12	56
**MCP-4**	5	1
**M-CSF**	9	14
**M-CSF R**	5	23,009
**MDC**	8	28
**MEC (CCL-28 / VIC)**	15	836
**MICA**	29	146
**MICB**	17	91
**MIF**	25	18
**MIG**	3	7
**MIP-1a**	5	17
**MIP-1b**	1	1
**MIP-1d**	65,279	53,169
**MIP-3a**	139	166
**MIP-3b**	121	16
**MMP-1**	263	352
**MMP-2**	86	37
**MMP-3**	1	10
**MMP-8**	1	1
**MMP-9**	4	1

**MMP-10**	6	28
**MMP-13**	1	25
**MPIF-1**	8	88
**MSP**	7	1
**NAP-2**	6	1
**NGF R**	13	53,169
**NRG-I-b1**	1	1
**NT-3**	29	255
**NT-4**	19	20
**OBS (Obstatin-C-terminus)**	55	114
**Oncostatin M (OSM)**	15	1
**Osteoprotegerin (OPG)**	49	53,626
**PARC**	39	1,184
**P-Cadherin**	40	53,627
**PDGF AA**	59	347
**PDGF AB**	51	256
**PDGF BB**	36	1
**PDGF R alpha**	33	1
**PDGF R beta**	1	1
**PECAM-1 (CD31)**	1	52
**PF-4**	30	95
**PIGF**	5	1
**Prolactin**	16	44
**P-selectin**	4	1
**RAGE**	21	142
**Rantes**	1	2
**SAA**	7	4
**SCF**	18	1
**SCF R**	14	11
**SDF-1a**	8	1
**SDF-1b**	5	1
**Shh-N**	7	4,115
**Siglec-5**	9	126
**Siglec-9**	19	1
**ST2 (IL-1R4)**	17	53,300
**TARC**	8	1
**TECK**	8	182
**TGF-a**	4	17
**TGF-beta 1**	161	654
**TGF-beta 2**	30	42
**TGF-beta 3**	35	1
**Tie-1**	56	53,027
**Tie-2**	23	49,197
**Timp-1**	24	1
**Timp-2**	12	60
**Timp-3**	10,617	27,745
**Timp-4**	34	184
**TNF-a**	26	8
**TNF-b**	8	33
**TNF-R I**	9	1
**TNF-R II**	5	10
**TPO**	5	4
**TRAIL**	1	50
**TRAIL R1**	1	53,211
**TRAIL R2**	1	53,348
**TRAIL R3**	1	3,498
**TRAIL R4**	14	53,543

**TRANCE**	1	2,021
**TREM-1**	1	59
**TROY**	1	358
**u PAR**	1	1
**ubiquitin+1**	1	50,906
**VCAM-1**	1	96
**VE- Cadherin**	1	5,036
**VEGF**	2	1,161
**VEGF R2**	11	51,783
**VEGF R3**	4	6,518
**VEGF-D**	5	135
**VEGI**	1	1
