# Supplementary material for: PreImplantation factor (PIF) detection in maternal circulation in early pregnancy correlates with live birth (bovine model)
Source: Reprod Biol Endocrinol. 2013 Nov 15;11:105. doi: 10.1186/1477-7827-11-105 (PMC3842769; doi:10.1186/1477-7827-11-105)
Supplement: Additional file 3: Figure S2 — PIF source in cow placenta. Description: The expression of PIF in first trimester cow placental tissue samples was analyzed using immunohistochemistry at different magnifications. Anti-PIF-mAb binding was tested at different concentrations, 50–200 μg/ml and results were compared to samples tested with an antibody against a mouse non immune serum (negative control). Results show that optimal anti-PIF-mAb (50 μg/ml) detects PIF within the placenta and the effect was dose dependent (a,b,c) in contrast to control antibody that failed to bind (d). [file 1477-7827-11-105-S3.pdf]

Antibody was tested on bovine placenta at a concentration of 50ug/ml

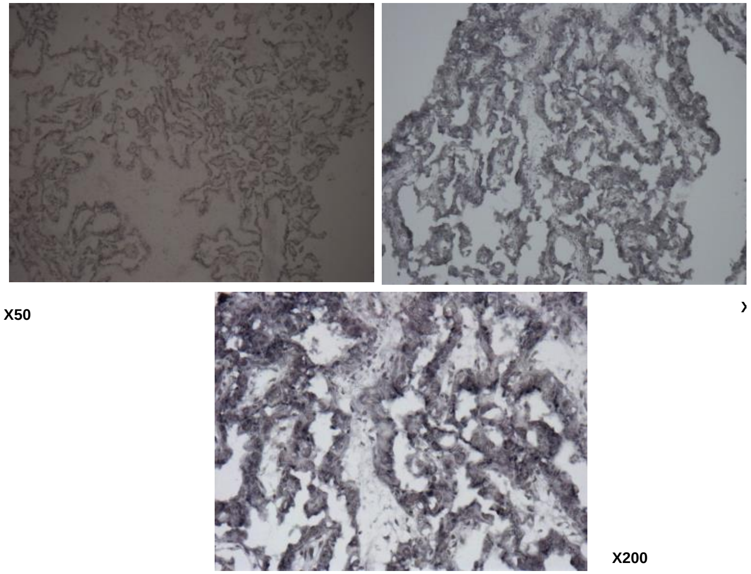

Antibody was tested on bovine placenta at a concentration of 100ug/ml

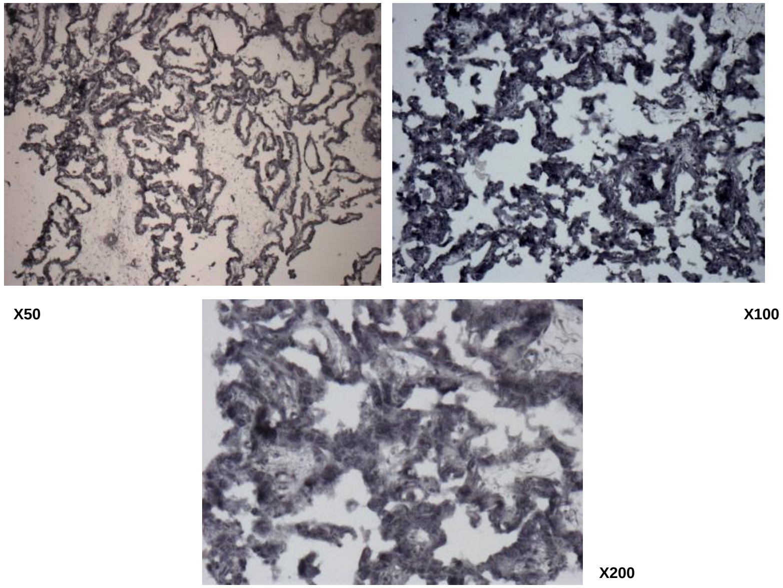

Antibody was tested on bovine placenta at a concentration of 200ug/ml

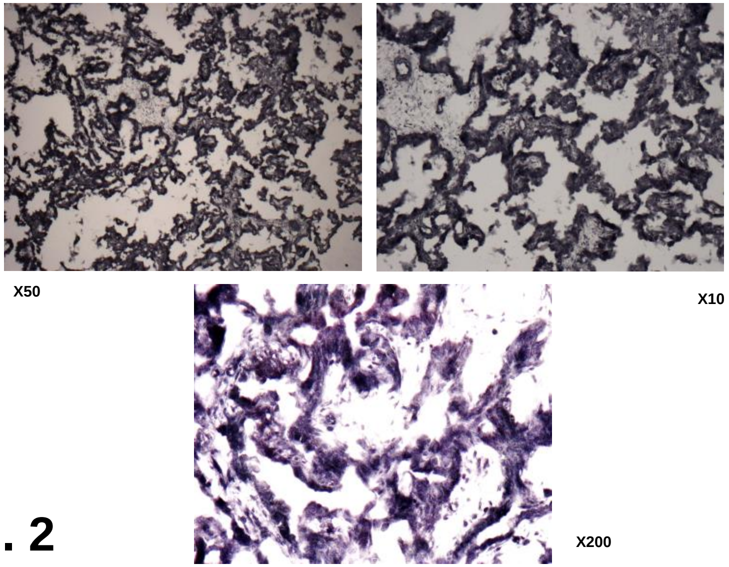

Negative control

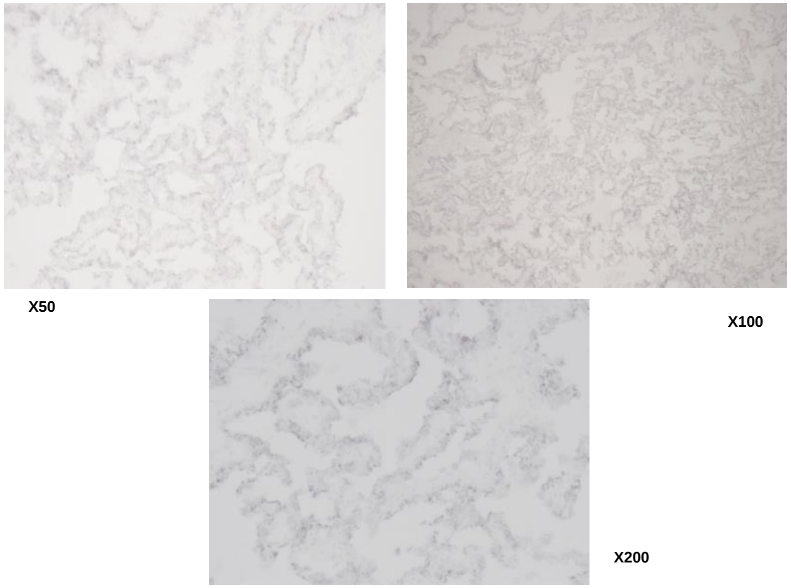

Suppl Fig. 2
